# Functional Role of NRF2 in Cervical Carcinogenesis

**DOI:** 10.1371/journal.pone.0133876

**Published:** 2015-08-06

**Authors:** Jun-Qi Ma, Hatila Tuersun, Shu-Juan Jiao, Jian-He Zheng, Jing-Bao xiao, Ayshamgul Hasim

**Affiliations:** 1 Department of Gynecology, the First Affiliated Hospital of Xinjiang Medical University, Urumqi, China; 2 Department of Pathology of Medical University of Xinjiang, Urumqi, China; University of Navarra, SPAIN

## Abstract

Nuclear factor erythroid-2-related factor 2 (NFE2L2) is a transcription factor associated with resistance to chemotherapy and increased tumor growth. NRF2 is repressed by the inhibitor Keap1. The Keap1-NRF2 pathway is dysfunctional in multiple tumor types. Among Uighur women, the incidence of cervical squamous cell carcinoma (CSCC) and cervical intraepithelial neoplasia (CIN) was associated with elevated nuclear expression of NRF2 and decreased cytoplasmic expression of Keap1. Up-regulation of nuclear NRF2 was significantly associated with reduced cytoplasmic Keap1 expression. NRF2 positivity and Keap1 negativity were frequently associated with more advanced tumors (i.e., higher histological grade, lymph node involvement, and higher tumor stages) (p<0.05 for all). Methylated CpG islands in the Keap1 gene promoter in cervical cancer tissue were identified using MassARRAY. Moreover, promoter hypermethylation of this gene was significantly associated with decreased protein expression and increased nuclear NRF2 expression in cervical cancer tissues. Overexpression and knockdown of NRF2 in CSCC cell lines showed that NRF2 promotes proliferation, inhibits apoptosis, and enhances migration and invasion. These studies support the concept that epigenetic changes regulate expression of Keap1 in cervical cancer tissues. The association of NRF2 expression with aggressive tumor behavior suggests that NRF2 may be a marker of poor prognosis in patients with cervical cancer.

## Introduction

Cervical cancer is a major global health problem. It is the second most commonly diagnosed cancer among women, with more than 500,000 new cases reported each year. More than half of these cases will end in death [1]. The introduction of the Papanicolaou (Pap) smear as a screening procedure for cervical cancer has reduced the incidence of this disease in developed countries. However, there is inadequate support for patients with cervical cancer in many developing countries. Approximately 80% of cervical cancer cases occur in these countries [2]. There are high morbidity (590/100,000) and mortality rates from cervical squamous cell carcinoma (CSCC) among Uighur women, especially in the south of Xinjiang. The incidence of cervical cancer among Uighur women is four times higher than that of China (138/100,000) [3].

Human papilloma virus (HPV) infection is the major cause of cervical cancer. Cervical epithelial tissues are exposed to oxidative stress (OS), which promotes the development of persistent, chronic viral infections. The integration of the viral genome in the host cell produces genetic rearrangements, genomic instability, and increased risk of neoplastic transformation [4–5]. Fortunately, cervical epithelial tissue has an antioxidant system consisting of the transcription factor nuclear factor erythroid 2-related factor 2 (NRF2). NRF2 maintains redox balance in the cell by controlling gene transcription. This potent transcriptional activator recognizes and binds to the antioxidant response element (ARE) in target gene promoters. The ARE has a conserved basic leucine zipper (bZIP) structure and is a member of the Cap ‘N’ Collar (CNC) family. ARE transactivation induces the expression of genes that protect cells in response to oxidative and electrophilic stressors [6]. Kelch-like ECH-associated protein 1 (Keap1) mediates ubiquitination and degradation of factors that are involved in cell survival and apoptosis. Keap1 regulates these activities in conditions of oxidative stress and inhibits NRF2 activity through ubiquitin-dependent degradation [7]. In normal conditions, NRF2 is expressed at low basal levels, because Keap1 maintains constant turnover of NRF2 through ubiquitination and subsequent degradation. Upon exposure to oxidant or xenobiotic stress, Keap1 is inactivated. Cysteine residues in Keap1 are modified [8], delaying degradation. NRF2 then forms a heterodimer with a small Maf family protein and translocates to the nucleus to bind to the ARE. This leads to the induction of many cytoprotective genes [9–11]. Thus, Keap1 appears to function as a tumor suppressor. Loss of Keap1 function increases tumorigenesis. KEAP1 gene hypermethylation in malignant gliomas, breast cancers, and colorectal cancers is associated with loss of function [12–14]. However, the methylation status of *KEAP1* in cervical cancer is unknown. Emerging data suggests that overexpression of NRF2 is associated with cancer development and progression [15–18]. NRF2 protects normal cells from transformation but also promotes proliferation and survival. However, the role of NRF2 in cervical cancer remains unclear.

We examined the methylation status of *KEAP1* in 16 surgically excised CSCC tissue samples and matched normal cervical epithelial tissues. In addition, we evaluated the expression of NRF2 and Keap1 in 89 cases of cervical cancer and examined associations with pathologic features and clinical outcomes. Finally, we examined the functional role of NRF2 in cervical SiHa cells.

## Materials and Methods

### Ethics Statement

All patients and controls provided written informed consent, and we received study approval from the ethics committee of the First Affiliated Hospital of Xinjiang Medical University.

### Patient Samples

We obtained cervical tissue specimens from Uighur women with CSCC and from those who did not have cervical diseases but received hysterectomies in the Department of Gynecology at the First Affiliated Hospital in Medical University of Xinjiang. All cancers were staged in accordance with the criteria established by the International Federation of Gynecology and Obstetrics (FIGO). Formalin-fixed, paraffin-embedded (FFPE) tissues (n = 89) were obtained from the Department of Pathology. FFPE specimens or fresh-frozen cervical tissues were collected during an initial outpatient visit, during gynecologic examination, or after a surgical procedure involving general anesthesia. Tumor samples were collected within 30 minutes (min) of surgical resection. None of the patients received chemotherapy or radiation prior to surgery. After evaluation by a pathologist, tumor tissues were immediately frozen in liquid nitrogen and stored at -80°C. Haematoxylin and eosin staining was also performed to confirm the diagnosis and to analyze pathological grades, metastasis, and tumor cell content. Seventy percent of all tumor samples were composed of tumor cells free of necrosis.

Patients included 54 FIGO stage I B and 35 FIGO stage IIB. There were 38 well-differentiated cases, 23 moderately differentiated and 28 poorly differentiated tumors. Lymph node metastasis was documented in 31 patients. Forty-six patients with CIN I-II were selected for this study. The median age of patients with cervical cancer was 49.5 years (IQ range 28–65.5 years). Control tissues (n = 66) were from patients who did not have cervical lesions or cancer but had hysterectomies for other reasons (i.e., fibroids, prolaps uteri, adenomyosis, or a combination of fibroids with prolaps uteri) during the same time period.

Thirty-two biopsies, including 16 cases of CSCC and 16 matched cases of normal epithelia located 5 cm from the tumor were collected within 30 min of resection and stored at -80°C until gene methylation analysis.

### Immunohistochemistry (IHC)

IHC staining was performed with an anti-Keap1 rat monoclonal antibody (1:5,000) and an anti-human NRF2 mouse monoclonal antibody (1:300 Abcam, Cambridge, MA, USA). Sections (3-mm-thick) were cut from paraffin-embedded tissue blocks. Samples were dewaxed in xylene and rehydrated in alcohol and distilled water. Antigen retrieval was then performed by heating samples for 15 min at 95°C in citrate buffer (pH 6.0). Samples were cooled to room temperature and incubated in 3% hydrogen peroxide to quench peroxidase activity. After incubating at 4°C overnight in primary antibody and washing with Tris buffer, biotin-labeled secondary antibody was added for 15 min followed by streptavidin peroxidase for 15 minutes. After eluting with PBS, diaminobenzidine and haematoxylin counterstaining were performed.

Two pathologists evaluated the percentage and intensity of staining in tumor cells in a blinded manner. The pathologists reached a consensus number for each tumor sample. Nuclear NRF2 and cytoplasmic Keap1 were quantified according to intensity (0, 1+, 2+, or 3+) and percentage (0%–100%) of staining. An IHC expression score was calculated by multiplying intensity and percentage (range, 0–300). Positive nuclear NRF2 staining was defined as >0. Low or absent cytoplasmic Keap1 was defined as <150, which was the mean score of all CSCC cases. Nuclear NRF2 is thought to be biologically active [19].

### Quantitative DNA methylation analysis

Genomic DNA was extracted with a QIAamp DNA Mini Kit (QIAGEN, Valencia, CA). MassARRAY (Sequenom, San Diego, CA, USA) was performed for quantitative detection of methylated DNA. The human *KEAP1* DNA sequence was obtained from the NCBI human genome database. We used online Methprimer software to identify CpG islands around the transcription start site of the *KEAP1* gene. *KEAP1*-gene specific primer pairs were designed with the Sequenom Standard EpiPanel (forward: 5'-aggaagagag TTAGTTATTTAGGAGGTTGT-3'; reverse: 5’- cagtaatacgactcactatagg gagaaggct AACCCCCCTCTCA-3’) [20]. PCR samples included 10 ng bisulfite-treated DNA, 200 mM dNTPs, 0.2 U Hot Start Taq DNA polymerase (QIAGEN), and 0.2 mM forward and reverse primers in a total volume of 5 ll. PCR cycles were as follows: hot start at 94°C for 15 min, denaturation at 94°C for 20 seconds, annealing at 56°C for 30 seconds, extension at 72°C for 1 min (45 cycles), and a final incubation at 72°C for 3 minutes. We added 2 ml premix with 0.3 U shrimp alkaline phosphate (SAP; Sequenom) to dephosphorylate unincorporated dNTPs and then incubated at 37°C for 40 mi. SAP was heat-inactivated for 5 min at 85°C. In vitro transcription was performed following SAP treatment, using 2 ml PCR product. RNase A cleavage was used for reverse reaction, as suggested by the manufacturer (Sequenom). After conditioning, samples were spotted on a 384-pad Spectro-CHIP (Sequenom) with a MassARRAY nanodispenser (Samsung, Irvine, CA, USA). Spectral acquisition occurred with a MassARRAY analyzer compact MALDI-TOF mass spectrometer (Sequenom). Methylation analyses were performed with the EpiTYPER application (Sequenom) to quantify each CpG site or aggregates of multiple CpG sites.

### Cell culture and transfections

SiHa cells (ATCC; Manassas, VA, USA), a human cervical squamous cell carcinoma cell line, were cultured in RPMI 1640 plus 10% calf serum and 1% penicillin/streptomycin in a 5% CO2 humidified incubator at 37°C. SiHa cells were seeded in six-well plates and grown to 60%-80% confluence. NRF2 in the eukaryotic expression vector pcDNA3.1 (5- TCCGCTCGAGATGA TGGACTTGGAGCTGCC-3, antisense 5- ATGGGGTACCGAGTTTTTCTTAACATCTGGC-3), NRF2 inhibitor (10620318–267435 A03 / 10620318–267435 A05), and the scrambled sequence (CCAACCAGUUGACAGUGAACUCAUU / CAAACUGACAGAAGUUGACAA UUAU) were synthesized by Invitrogen (SHANGHAI, CN). Transfection complexes were formed with Lipofectamine RNAiMAX (Invitrogen, CA, USA) in Opti-MEMI (Invitrogen, CA, USA) according to manufacturer guidelines. Negative controls were cultured in normal conditions. All transfections were performed in triplicate. Cell proliferation was determined by counting cells 24, 48, and 72 hours (h) after transfection. RNA and protein were extracted 48 h or 72 h, respectively, after transfection.

### RNA isolation and qRT-PCR

We isolated total RNA using Trizol reagent (Invitrogen, CA, USA) per manufacturer’s instructions. RNA was reverse transcribed into cDNA using the Prime-Script one-step qRT-PCR kit (C28025-032, Invitrogen). qRT-PCR Forward primer is 5’-TCAGCGACGGA AAGAGTATGA-3’.reverse primer is 5’-CCACTGGTTTCTGACTGGATGT-3’.All samples used SYBR Select Master Mix (Applied Biosystems, USA). We evaluated t-Actin expression for normalization. Relative gene expression was determined with the comparative delta-delta CT method (2 -△△Ct). Reaction mixtures for NRF2 analyses were incubated at 95, USA). We evaluated t-Actin expression for normalization. Relative gor 1 minute. We evaluated β-actin at 95°C for 10 min and 40 cycles at 95°C for 15 seconds followed by 55°C for 1 minute.

### Protein isolation and western blotting

Protease inhibitors (Boster, Wuhan, China) were added to cell lysates, which were maintained on ice for 20 minutes. Lysates were then centrifuged at 12,000 rpm for 10 min at 4°C. Samples (50 μg) were boiled for 5 min in sample buffer and then separated on 12% gels by SDS-PAGE. Gels were transferred onto nitrocellulose membranes and blocked for 1 h in 5% skim milk at room temperature with shaking. A primary antibody against NRF2 (Abcam, USA) or β-Actin (Sangon, Shanghai, China) was added overnight to blots at 4°C. Blots were washed in PBS-Tween three times, after which the secondary antibody (horseradish peroxidase-conjugated goat anti-rabbit immunoglobulin G; Thermo, IL, USA) was added at room temperature for 2 hours. Chemiluminescent substrate (Thermo, IL, USA) was added to visualize bands. Quantity One software was used to quantify the intensity of each band and was normalized to the intensity of the internal control β-Actin. Results were expressed as fold changes normalized to control values.

### Analyses of cell cycle and apoptotic changes by flow cytometry

SiHa cells were seeded in six-well culture plates at a density of 5×10^4^ cells/well in RPMI 1640 plus 10% calf serum and 1% penicillin/streptomycin. High-fucose-content (HFC) polysaccharide (50, 100, 200, or 250 μg/mL) was added for 1 h followed by the treatment with 300 μM H_2_O_2_ for varying time points (0–24 h). Cell cycle distributions were examined by measuring PI-fluorescence with a BD FACS Calibur flow cytometer (Becton Dickinson, San Jose, CA, USA) through an FL-2 filter (585 nm). We recorded 1×10^4^ events per sample. Data were analyzed with Cell Quest.

Annexin V staining was performed to evaluate apoptosis. Control and treated SiHa cells were added at 5×10^5^ cells/mL in binding buffer (10 mM HEPES [(4-(2-hydroxyethyl)-1-piperazineethanesulfonic acid] [pH 7.4], 140 mM NaCl, 2.5 mM CaCl2). FITC-annexin V (10 μl) in 190 μl of cell suspension was incubated for 10 min at room temperature. Cell mixtures were centrifuged and resuspended in 190 μl binding buffer, and 10 μl PI (1 mg/mL) solution was added. Cells were acquired on a FACS Calibur flow cytometer at 1×10^4^ events per sample. Necrotic cells were defined as positive for both PI and annexin V and were excluded from further analysis.

### Transwell migration and invasion assays

Migration and invasion assays were performed as previously described. Migration was evaluated in Transwell cell culture chambers with 6.5-mm-diameter polycarbonate membrane filters containing 8-μm pores (Corning, NY, USA). Cells were added in 100 ml serum-free media to the upper chamber. The lower chamber contained 600 ml culture media with 10% calf serum. After 10 h at 37°C, cells were removed from the upper surface of the membrane with a cotton swab. Filters were fixed in methanol for 20 min and stained with Giemsa solution for 30 minutes. We then counted the number of cells that had migrated. Five random fields (Nikon ECLIPSE TS100) were counted per well, and the mean was calculated. The membrane of the upper chamber of the transwell was pre-coated with 100 ml of a 1mg/ml solution of Matrigel (BD, USA).

### Statistical analysis

Statistical analyses were determined using SPSS Version 17. P values were two-sided, and the significance level was P<0.05. Values were expressed as means ± SEM. Statistical analyses were conducted using the two-tailed Student’s t-test upon verification of the assumptions. Mann-Whitney test was used to test continuous variables for differences in NRF2 IHC scores between tumor and normal tissues. In addition, we performed Spearman’s tests for correlations.

## Results

### NRF2 and Keap1 expression in female Uighur patients with cervical cancer or CIN

**Fig 1:**
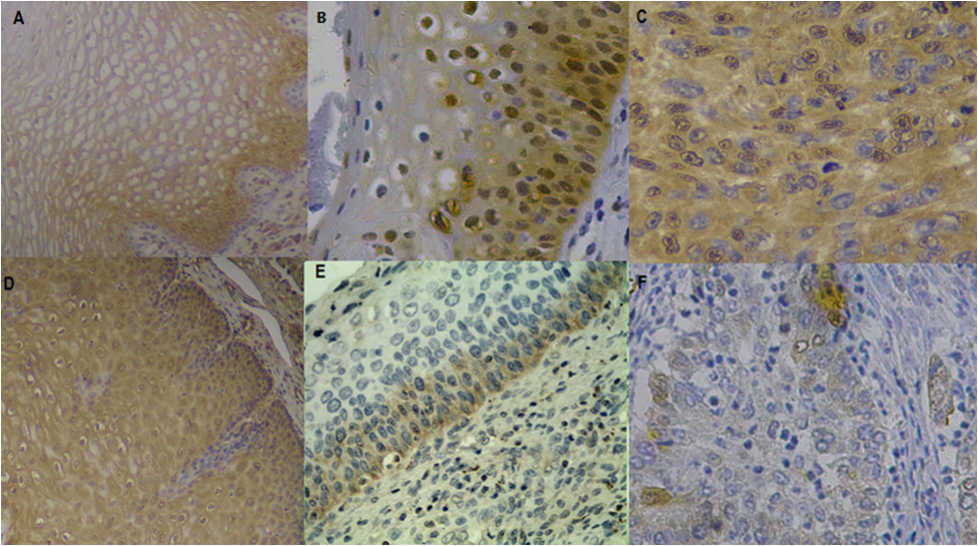
Detection of NRF2 and Keap1 protein expression assessed by immunohistochemical staining in representative specimens of normal cervical epithelia, CIN and CSCC, respectively. A: Expression of NRF2 in normal cervical epithelia with weak cytoplasm staining; B: Moderate expression of NRF2 protein in CIN tissue; C: Strong nucleus expression of NRF2 proteins in CSCC tissue; D: Expression of Keap1 in normal cervical epithelia with Strong cytoplasm staining; E: Moderate expression of Keap1 protein in CIN tissue; F: weak expression of Keap1 proteins in CSCC tissue. (original magnification, × 200).

Antibodies were tested on formalin-fixed, paraffin-embedded, normal cervical tissues and CSCC. Representative IHC images for NRF2 and Keap1 are shown in Fig 1. NRF2 was primarily localized in the nuclei of CSCC and CIN cells. By contrast, NRF2 was mainly localized in the cytoplasm of normal cervical epithelial cells. Nuclear expression of NRF2 was significantly increased compared with that of cervical intraepithelial neoplasia and normal cervical epithelium (P<0.05). Keap1 expression was significantly decreased in the cytoplasm of cancer cells compared with that of normal cervical epithelial cells (P<0.05).

We correlated expression of NRF2 and Keap1 with clinical data in 89 patients with CSCC. The Mann-Whitney test was used to test differences in IHC scores between tumor and normal cervical epithelium for continuous variables. Increased expression of NRF2 was significantly associated with positive lymph node metastasis and poor differentiation (Table 1). Keap1 staining correlated with tumor stage (FIGO stage I B to IIB), lymph node status (N0 to N1), and pathological grade (GI to GIII) (Table 1). We determined the association between cytoplasmic Keap1 and nuclear NRF2 expression in patients with CSCC. The Spearman rank correlation coefficient test results showed that reduced cytoplasmic Keap1 was associated with nuclear NRF2 (P = 0.021, R = 0.249).

**Table 1:** Statistical analysis of NRF2 and Keap1expression and clinicopathologic factors in CIN and cervical cancer.

Characteristics	N	NRF2(-)	NRF2 (+)		Keap1 (-)	Keap1 (+)	*P*
Normal mucous epithelia	66	48	18		28	38	0
CINⅡ-Ⅲ	46	20	26		27	19	
CSCC	89	30	59	0	69	20	
Differentiation							
Well	38	18	20		28	10	0
Moderate	23	8	15		18	5	
Poor	28	4	24	0.002	23	5	
L/N metastasis							
Negative	58	25	33		44	14	
Positive	31	5	26	0.004	25	6	0.004
FIGO Stage							
≤ I B	54	23	31		46	8	
> IIB	35	7	28	0.019	23	12	0.032

### *KEAP1* DNA methylation in cervical cancer samples from female Uighur patients

We evaluated levels of KEAP1 methylation in normal tissues and tumor specimens using a Sequenom MassARRAY platform. There were 12 CpG sites in which the methylation levels were significantly higher (P<0.05) in CSCC (0.117 ± 0.057) than in normal controls (0.058 ± 0.031). Methylation at single CpG sites showed significant differences between CSCC and normal cervical epithelia at CpG1, CpG3, CpG6, and CpG10 (Table 2), It is possible that these are binding sites for proteins that regulate Keap1 expression. Further analyze the correlation between Keap1 expressions with its DNA methylation in CSCC tissue; the results showed an inverse correlation of altered CpG island methylation of Keap1 with changes in protein expression (Table 3).

**Table 2:** Quantitative analysis of Keap1 gene single CpG site methylation by Sequenom MassARRAY.

CpG site	Tumor tissues Methylation levels ($\bar{x}\pm s$)	normal adjacent tissues Methylation levels ($\bar{x}\pm s$)	t	*P*
Keap1 _CpG_1	0.117±0.018	0.037±0.018	3.384	0.035
Keap1 _CpG_2	0.089±0.082	0.083±0.053	-0.066	0.948
Keap1 _CpG_3	0.121±0.011	0.030±0.018	7.124	0.001
Keap1 _CpG_4	0.155±0.053	0.103±0.065	1.421	0.176
Keap1 _CpG_5	0.173±0.016	0.122±0.022	1.124	0.091
Keap1 _CpG_6	0.052±0.086	0.016±0.390	3.713	0.013
Keap1 _CpG_7	0.069±0.013	0.048±0.016	0.289	0.775
Keap1 _CpG_8	0.005±0.013	0.002±0.008	-0.892	0.382
Keap1 _CpG_9	0.091±0.076	0.067±0.021	1.564	0.073
Keap1 _CpG_10	0.126±0.019	0.094±0.015	3.597	0.027
Keap1 _CpG_11.12	0.155±0.053	0.043±0.065	1.421	0.176

**Table 3:** Statistical analysis of Keap1 expression and Keap1 methylation level s in CSCC.

Keap1 protein expression	Keap1 methylation level(mean ± SD)	F	*P*
−	0.117 ± 0.057		
+	0.058 ± 0.031	-2.211	0.042

### NRF2 promotes cell proliferation and apoptosis in SiHa cells

**Fig 2:**
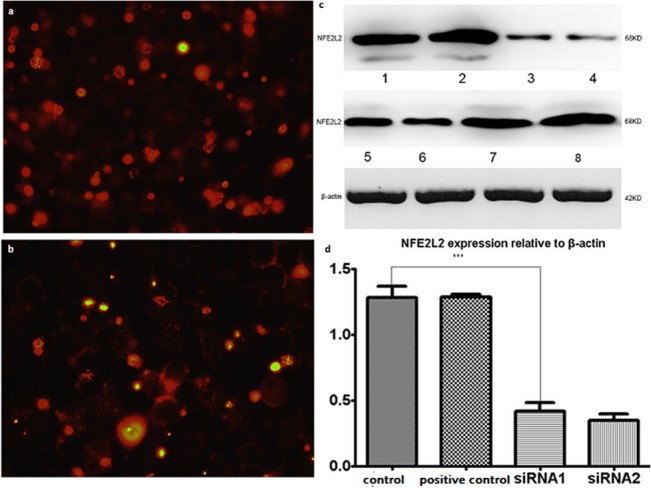
The detection of NRF2 protein transfection with mimics and inhibitor. Morphology of transfected Siha cells for 48 h under microscopy (magnification ×200). A. Transfection with Short interfering RNA (SiRNA); B. transfection with mimics; C. The levels of NRF2 protein detected by Western blotting after transfection for 72 h.1 and 2 were normal control; 3 and 4 were Knockdown group; 5 and 6 were normal control; 7 and 8 were overexpression group; D The relative expression of NRF2 was displayed, which normalized to b-tubulin. There is a statistically significant difference between the group transfected with NRF2 mimics, NRF2 inhibitor and normal control.

NRF2-specific short hairpin (shRNA) or a full-length human NRF2 was successfully transfected into SiHa cells. The transfection efficiency was as high as 88.9%. Transfection efficiency of SiHa cells expressing NRF2-shRNA was assessed by flow cytometry (Becton–Dickinson, Franklin Lakes, NJ, USA). Expression of NRF2 was detected by real-time quantitative PCR and Western blotting. Both NRF2 mRNA and protein levels were significantly decreased after transfecting NRF2-shRNA compared with the vector control and normal groups. Conversely, NRF2 expression was significantly increased after transfecting pCDNA3.1·NRF2 (Fig 2).

**Fig 3:**
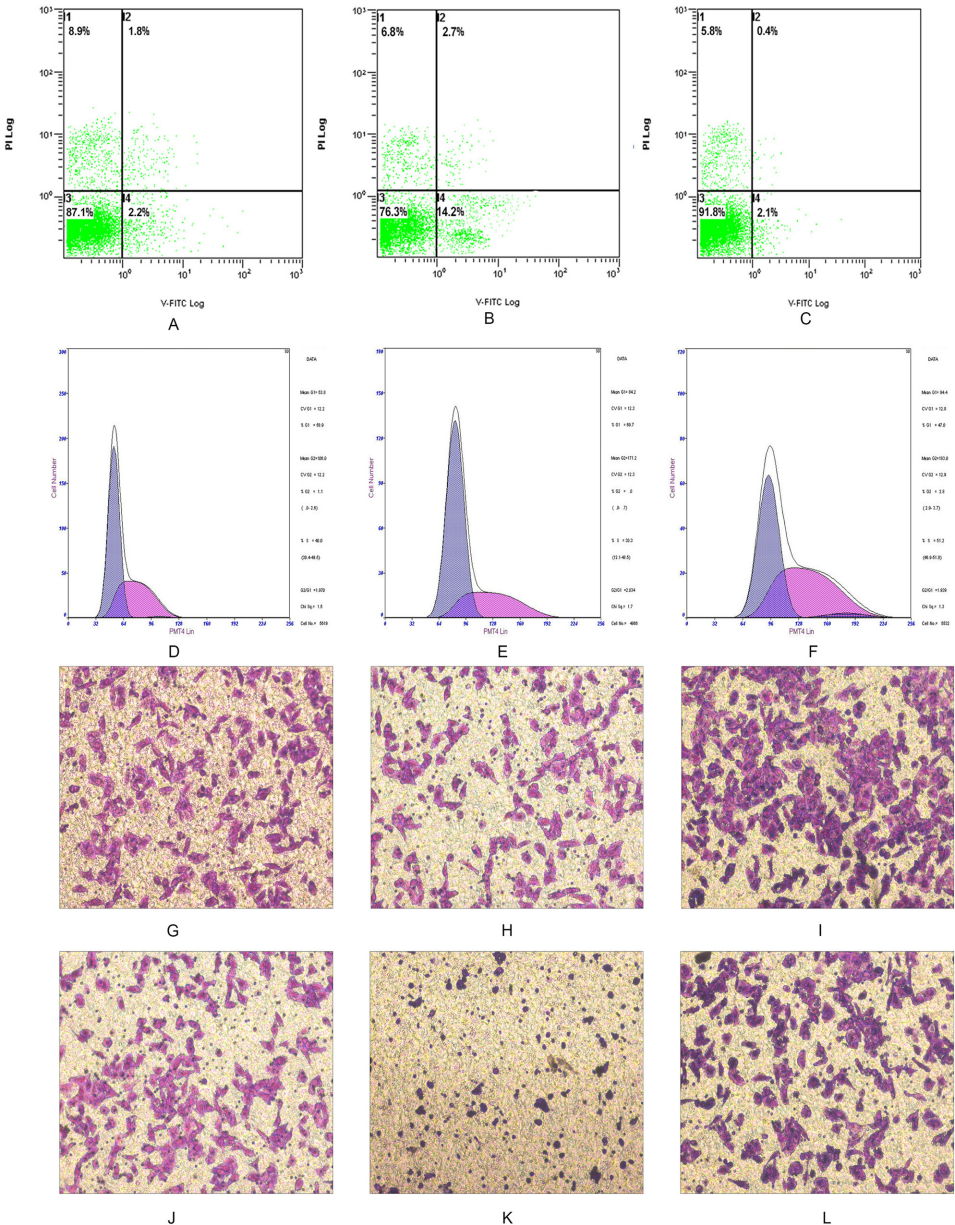
NRF2 positively modulates CSCC cellular malignant phenotypes. A, D, G and J: Cell apoptosis, Proliferation, Migration and invasion in Siha cells, respectively (Normal controls). B, E, H and K: a Knockdown of NRF2 increased cell apoptosis, decreased cell proliferation, migration and invasion, which significantly decreased malignant phenotypes of Siha cells. C, F, I and L: Overexpression of NRF2 sharply decreased cell apoptosis, increased cell proliferation, migration and invasion, which significantly enhanced cell proliferation and migration in Siha cell line. All experiments were performed at least three times.

Using flow cytometry analysis investigates apoptosis and proliferation rates of Siha cells after altered NRF2 expression (Fig 3). The percentage of SiHa cells in G0/G1 phase significant increased (74.90%±4.17%) 48 h after NRF2 knockdown compared with the percentage of control cells in G0/G1 (53.97%±2.89%). The percentage of NRF2 shRNA-transfected cells in S phase was significantly decreased (21.87%±4.67%) compared with of control (41.97%±3.70%). Over-expression of NRF2 significantly increased the percentage of cells in G0/G1 at 48 h (40.33%±1.25%) compared with that of control (53.57%±2.86%). The percentage of NRF2-transfected cells in S phase increased (57.37%±1.86%) compared with control (44.50%±2.35%) (Table 4). These results suggest that Overexpression of NRF2 increased the basal proliferation rates and promotes DNA replication of the Siha cell lines (Table 5). There were 14.13%±0.51% of SiHa cells that demonstrated apoptotic changes 48 h after NRF2 knockdown; this was a significant increase compared with control (2.68%±0.38%, Table 6). By contrast, over-expression of NRF2 did not significantly change apoptosis (3.67%±0.35% compared with 3.60%±0.50% in control; P>0.05).

**Table 4:** Changing Siha cells cycle after NFE2L2 siRNA vector transfect 48 hours ($\bar{x}\pm s$,n = 3).

	G0/G1(%)	S(%)	G2/M(%)
Control	54.40±3.91	42.23±3.12	3.37±2.87
Negative control	53.97±2.89	41.97±3.70	4.10±0.87
NFE2L2 A07	68.07±3.45^△^	30.43±0.91^△^	1.50±2.60
NFE2L2 A05	74.90±4.17^△^	21.87±4.67^△^	3.27±1.86

note: △compared with control group, P<0.01

**Table 5:** Changing Siha cells cycle after PcDNA3.1NFE2L2 vector transfect 48 hours ($\bar{x}\pm s$,n = 3).

	G0/G1(%)	S(%)	G2/M(%)
control	53.40±2.16	43.80±1.04	2.80±1.25
Negative control	53.57±2.86	44.50±2.35	1.93±0.51
PcDNA3.1NFE2L2	40.33±1.25^△^	57.37±1.86^△^	1.30±0.62

note: △compared with control group, P<0.01

**Table 6:** Changing apoptosis rate of Siha cell lines in response to altered NRF2 expression by transfect NFE2L2 siRNA vector after 48 hours ($\bar{x}\pm s$,n = 3).

	apoptosis rate of Siha cell(%)
control	2.93±0.67
Negative control	2.678±0.38
NFE2L2 A07	13.73±0.50^△▽^
NFE2L2 A05	14.13±0.51^△▽^

note: △compared with control group, P<0.01; ▽compared with Negative control group,P<0.001

### NRF2 induces cell migration and invasion in SiHa cells

**Fig 4:**
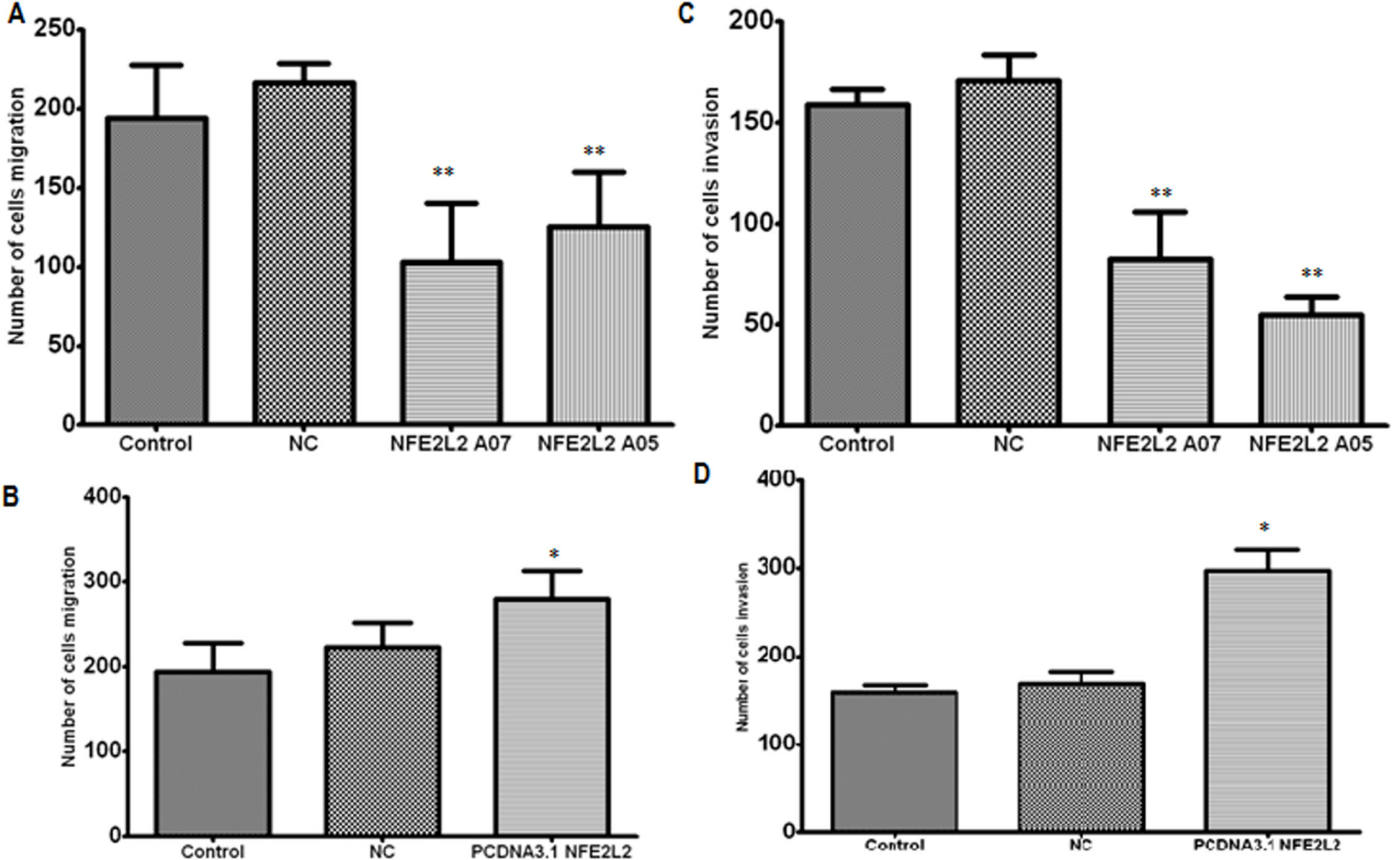
Effect of siRNA-expressing vectors and over-expressing vectors on the cell migration and invasion of Siha cells following transfection. For overexpression studies, NRF2 was overexpressed using a pcDNA3.1 vector and inhibition of NRF2 was achieved using siRNA vectors. A and B: regulates migration of Siha cells; C and D: regulates invasion of Siha cells. *P < 0.05; **P < 0.01.

Invasive growth is an important biological characteristic of malignant cancer cells. To investigate the role of NRF2 in cell motility, we performed a Transwell assay in Siha cells. The results show that Cell migration abilities was enhanced after NRF2 over-expression compared with control, and Siha cells with reduced expression of NRF2 were inhibited the migration ability. Overexpression of NRF2 increased the invasive abilities of Siha cells. As expected, Siha cells with reduced expression of NRF2 were less invasive compared with control cells (Fig 4).These results suggest that NRF2 promotes migration and invasion in SiHa cells.

## Discussion

The Keap1 and NRF2 pathway is a critical regulator of cellular responses to oxidative and electrophilic stressors [21]. Keap1/NRF2 protects normal cells from carcinogenesis but also promotes survival of transformed cells in unfavorable conditions [22]. This is the first report of increased Keap1/NRF2 signaling as a result of *KEAP1* hypermethylation in cervical cancer. In addition, this is the first report to show an association between NRF2/Keap1 staining and clinicopathological features in cervical cancer.

Keap1 binds to and sequesters NRF2 in the cytoplasm, preventing rapid degradation of NRF2. NRF2 translocates to the nucleus, inducing transcription of downstream cytoprotective genes. However, Keap1 has also been shown to be dysfunctional in non-small cell lung carcinomas that have elevated levels of NRF2 [23]. Thus, nuclear translocation of NRF2 may occur through Keap1-dependent or-independent pathways. We demonstrated that Keap1 was frequently hypermethylated with reduced expression in cervical cancer cells. Thus, the NRF2/Keap1 system may be dysregulated in human cervical cancers. Further, nuclear translocation of NRF2 may occur through a Keap1-independent pathway in cervical cancers. Constitutive NRF2 activation due to Keap1 dysfunction and hypermethylation has been reported [24]. The frequency of *KEAP1* promoter hypermethylation varies among tumor types. The highest frequency of promoter hypermethylation was found in malignant gliomas (70%) [14], non-small cell lung cancer (50%)[21], colorectal carcinoma (53%), and breast cancer (51%)[16]. In breast cancer, methylation was more frequent in ER-positive/HER2-negative tumors (66.7%) as compared with triple-negative breast cancers (35%). IHC studies in lung cancer, malignant gliomas, and breast cancer demonstrate a high frequency of Keap1 downregulation and NRF2 over-expression. Thus, our results and those of others suggest that epigenetic mechanisms regulate Keap1 expression. Further, dysregulation of Keap1 may play a role in carcinogenesis.

In the current study, NRF2 protein levels were markedly elevated in cervical cancer cells. Up-regulation of nuclear NRF2 was significantly associated with reduced expression of cytoplasmic Keap1. These data suggest that persistent nuclear expression of NRF2 may increase the production of antioxidants. Therefore, cervical cancer cells with nuclear NRF2 may have increased malignant potential. Our findings are in accordance with those of Ma et al. [25], who reported that upstaging of cervical cancer increases nuclear levels of NRF2 and enhances the expression of downstream proteins involved in the antioxidant response.

We found that the NRF2 mediated-defense system closely correlated with advanced staging. There were significant correlations between NRF2 expression and several clinicopathological factors, such as tumor lymph node metastases, clinical stage, and tumor grade. Our findings are in accordance with those of Kawasaki et al. [16], who reported that expression of NRF2 in gastric cancer was significantly associated with differentiation, stage, and lymph node metastases. Thus, increased nuclear NRF2 may be a marker of poor prognosis in patients with cervical cancer.

We also investigated the functional role of NRF2 in a cervical cancer cell line. Proliferation of SiHa cells was inhibited, and apoptosis was significantly increased in cells transfected with shRNA-NRF2. Migration and invasion were decreased, whereas proliferation was enhanced after over-expression of NRF2. Cells were arrested in G1 phase after expression of NRF2. Apoptosis was significantly inhibited, and migration and invasion were enhanced after knockdown. NRF2 protects tumor cells but also promotes oncogenesis. Mitsuishi et al. showed that NRF2 causes glucose and glutamine to enter anabolic pathways, enabling NRF2 to enhance metabolic activity, growth, and proliferation [26–27]. Oncogenes, such as K-Ras, B-Raf, and Myc increase transactivation of NRF2, which reduces endogenous ROS levels. These events may promote tumorigenesis [28]. NRF2 also upregulates the transcription of anti-apoptotic proteins, such as Bcl-2 and Bcl-xL, suppressing apoptosis, and increasing survival and drug resistance in cancer cells [29–30]. By contrast, knockdown of NRF2 in Casik cervical cancer cells, A549 non-small-cell lung cancer cells, and prostate cancer cells increased sensitivity to chemotherapeutic drugs and radiation [25,31–32]. These results greatly support a role for NRF2 in cancer cell survival and reduced response to anticancer chemotherapy and radiation therapy.

In summary, strong nuclear expression of NRF2 was significantly associated with reduced cytoplasmic Keap1 expression in cervical cancers due to hypermethylation. Our results, together with previous reports, [25] support hypermethylation of the *KEAP1* promoter region as a mechanism of Keap1 downregulation. Further, activation of NRF2 may increase cellular antioxidant and detoxification functions by inducing a wide variety of self-defense genes. Therefore, inhibition of NRF2 in combination with antineoplastic agents might be a promising therapeutic strategy in cervical cancer.
